# Size and shape variations of the bony components of sperm whale cochleae

**DOI:** 10.1038/srep46734

**Published:** 2017-04-25

**Authors:** Joseph G. Schnitzler, Bruno Frédérich, Sven Früchtnicht, Tobias Schaffeld, Johannes Baltzer, Andreas Ruser, Ursula Siebert

**Affiliations:** 1 Institute for Terrestrial and Aquatic Wildlife Research, University of Veterinary Medicine Hannover, Foundation, 25761 Büsum, Schleswig-Holstein, Germany; 2 Laboratoire de Morphologie Fonctionnelle et Evolutive, AFFISH Research Center, Université de Liège, B-4000 Liège, Belgium; 3 Laboratoire d’Océanologie, MARE Center, Université de Liège, B-4000 Liège, Belgium; 4 Röntgenpraxis Heide, Rungholtstr. 5 E-F, 25746 Heide, Schleswig-Holstein, Germany

## Abstract

Several mass strandings of sperm whales occurred in the North Sea during January and February 2016. Twelve animals were necropsied and sampled around 48 h after their discovery on German coasts of Schleswig Holstein. The present study aims to explore the morphological variation of the primary sensory organ of sperm whales, the left and right auditory system, using high-resolution computerised tomography imaging. We performed a quantitative analysis of size and shape of cochleae using landmark-based geometric morphometrics to reveal inter-individual anatomical variations. A hierarchical cluster analysis based on thirty-one external morphometric characters classified these 12 individuals in two stranding clusters. A relative amount of shape variation could be attributable to geographical differences among stranding locations and clusters. Our geometric data allowed the discrimination of distinct bachelor schools among sperm whales that stranded on German coasts. We argue that the cochleae are individually shaped, varying greatly in dimensions and that the intra-specific variation observed in the morphology of the cochleae may partially reflect their affiliation to their bachelor school. There are increasing concerns about the impact of noise on cetaceans and describing the auditory periphery of odontocetes is a key conservation issue to further assess the effect of noise pollution.

Sperm whales (*Physeter macrocephalus*), the largest toothed whales (Odontoceti), are highly pelagic animals and are normally found in deep oceanic waters. In some cases, however, individuals mistakenly wind up in the shallow, nutrient-poor North Sea during their migrations from the North Atlantic feeding grounds around the Norwegian shelf edge southwards to their breeding grounds around the Azores^1^. These long annual migrations are undertaken by males only, while females and calves stay close to the breeding grounds below 40° latitude throughout the year^2^,^3^,^4^.

During January and February 2016, several mass strandings of sperm whales occurred in the North Sea where thirty dead animals were observed along European coasts, amongst which sixteen beached on German coasts. The very shallow North Sea with a local coastline characterised by an intricate system of sand banks, mudflats, sandy islands and estuaries may have become a death trap because it is totally unsuitable for these deep-diving oceanic animals. Many theories have attempted to explain that seldom to rare phenomenon of sperm whale mass strandings, which probably result from complex interactions of physical (*e.g.* ocean currents, tides, geomagnetic anomalies, positive temperature anomalies and coastal configuration) and biological factors (*e.g.* social behaviour, food availability, echolocation or orientation failure and diseases)^5^,^6^,^7^,^8^,^9^,^10^,^11^. Mass strandings could also be related to military activities like underwater explosions, which have the potential to lead to injuries and hearing impairment due to the instantaneous onset, broad spectrum and high pressure of the blast^12^. Additionally, the usage of military sonars has been associated to have an effect on multiple cetacean species^13^. A mass stranding of sixteen whales of several cetacean species (Cuvier’s beaked whales, Blainville’s beaked whales, Minke whales, and a spotted dolphin) in the northern Bahamas was suspected to be a result of injuries caused by mid-frequency active sonar usage of naval ships^14^. Sperm whale strandings have been documented in the North Sea since the end of the 16^th^ century^15^ and occurred mostly in winter months between November and February in the period of male southward migration. Historically, all documented individuals were young males invariably with a body length between 12 to 18 m^15^.

Hearing abilities in an environment with low visual ranges seem crucial for the biology of whales. The auditory anatomy of toothed whales (Odontoceti) and baleen whales (Mysticeti) has been illustrated in many studies^16^,^17^,^18^,^19^,^20^,^21^,^22^,^23^,^24^. Whale ears are housed in two bulbous porcelainous bones: (1) the shell-like tympanic bulla forms the middle ear cavity that filters and amplifies sounds; (2) the periotic bone houses the inner ear operating as a mechano-electrical transducer of sound^20^,^24^.

The tympanic-periotic (T-P) bone complex of sperm whales shows several particularities shared among cetaceans. It is modestly dimensioned with a mass comparable to that of the killer whale (*Orcinus orca*), although the body size of sperm whales equals that of mysticetes^20^. The ears of most odontocetes tend to be separated from the skull by a suspension system of numerous ligamentous fibres that are generally considered to acoustically isolate the T-P bone complex to reduce mechanical sound propagation from skull vibrations^25^. However, the ears of sperm whales and beaked whales (Ziphiidae) retain a bony connection to the skull, which raises the possibility that a bone conduction mechanism may also exist in these two odontocete groups^25^. Finally, the posterior contact area between the tympanic and periotic bones is consolidated in a synostosis in sperm whales, while the T-P bone complex is relatively easy to take into its two parts in most other odontocetes^20^,^26^. Sperm whale ears are fully adapted to underwater hearing and have exceptional frequency discrimination abilities, with an estimated best hearing sensitivity that has a broad range, from 5 to 20 kHz^27^. The frequency sensitivity of the hearing system is evolutionarily related to habitat use and thus specific for most cetacean species^24^.

Little is known about the hearing capabilities and the functionality of acoustic pathways in *P. macrocephalus*, despite the increasing concerns about the impact of noise on cetaceans^27^,^28^,^29^,^30^. Describing the auditory periphery of odontocetes is a key conservation issue to further assess the effect of acoustic pollution^21^. To date, the intra-specific variation of auditory morphology in cetaceans is still poorly studied. No studies attempted to quantify form variation of cochlea (inner ear) within species, and no one investigated factors that may potentially explain such variations. The present study aims to explore the morphological variation of the auditory system in twelve sperm whales using high-resolution computerised tomography imaging. We performed a quantitative analysis of size and shape variation of cochleae using landmark-based geometric morphometrics to reveal inter-individual variations of the primary sensory organ of sperm whales. We examined if these variations were influenced by inter-individual differences related to growth, development and life history of the stranded whales to possibly reveal affiliation to separated groups.

## Results

### Specimens

**Figure 1:**
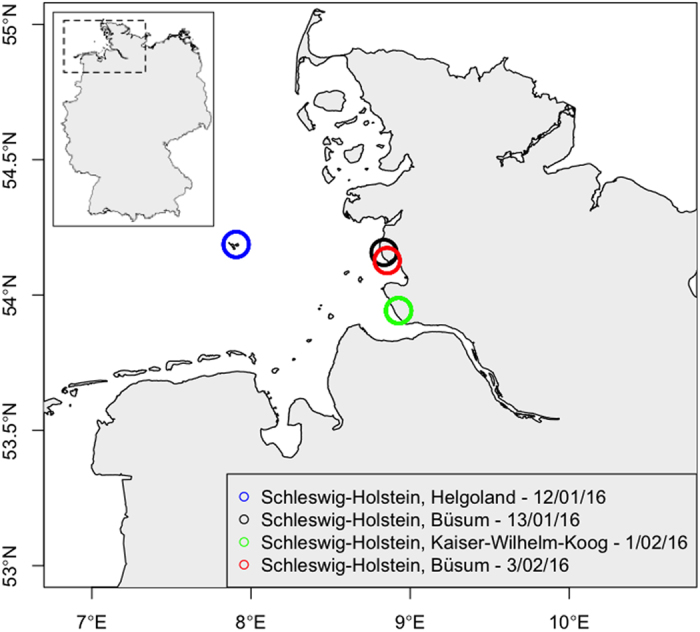
Sperm whale strandings recorded on German coasts of the North Sea during January and February 2016 (Map generated with ‘sp’ Package^59^,^60^ (version 1.2–3) and the ‘DEU_adm0.rds’ file (obtained from the GADM Global Administrative Database^61^) in R^58^(version 3.2.3)).

In a period of three weeks, twelve stranded sperm whales were dissected on the shores of Schleswig Holstein, Germany (Fig. 1). Based on their size and age, these individuals formed a homogenous group. They were young males aged between 10 and 15 years and showed a total body length of 10 to 12 meters (Table 1).

### Morphometric analysis of external characters

**Figure 2:**
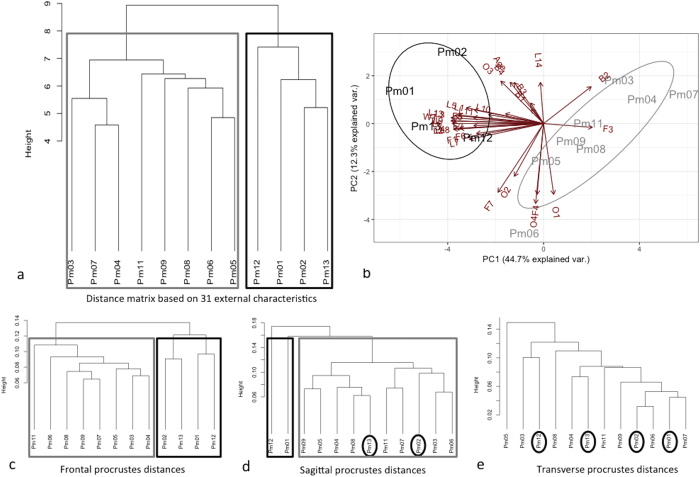
Discrimination of the 12 specimens of stranded sperm whales (**a**) Distance phenogram summarising the UPGMA clustering of 12 specimens of stranded sperm whales based on 31 external morphometric characters that were measured on each individual. The grey and black boxes illustrate the membership to the distinct stranding clusters. The cophenetic correlation is 0.76. (**b**) Projections of the 12 specimens of stranded sperm whales onto the first two principal components based on 31 external morphometric characters that were measured on each individual. (**c**,**d**,**e**) For confirmation purposes, we performed also UPGMA clusters based on the procrustes distances in frontal, sagittal and transverse view respectively.

Thirty-one morphometric characters were collected for all stranded sperm whales in order to characterize their external morphology and their condition (Table 2). We performed a hierarchical cluster analysis and Principal Component Analysis (PCA) based on these traits to reveal morphological similarity among individuals (Fig. 2a). The correlation between the original distances and the cophenetic distances was high (coefficient = 0.76), indicating that the dendrogram summarises the data appropriately. The unweighted pair-group method with arithmetic average algorithm (UPGMA) separates two main clusters: (1) a group of seven sperm whales that were found on 01/02 in Kaiser-Wilhelm-Koog with the specimen Pm03 that stranded earlier (13/01) in Büsum Süderpiep (Fig. 2a in grey) and (2) a group including the largest individuals (>11.4 m) with two animals that stranded on 12/01 on Helgoland and two others on 03/02 in Büsum (Fig. 2a in black). Within the first cluster, the individuals could then be separated on a size basis into the small specimens (<10.8 m; Pm03, Pm04 and Pm07) and the larger ones (10.8–11.4 m). The second group is rather heterogeneous and would partition Pm12 from the others (Fig. 2a). The discrimination between those two stranding clusters is mainly explained by PC1, which summarises size measurements (Fig. 2b). Most of the morphometric characters are highly related to the total length and the size of the fins, the PCA loadings being documented in Table 2.

### Morphology of sperm whale ear structures

**Figure 3:**
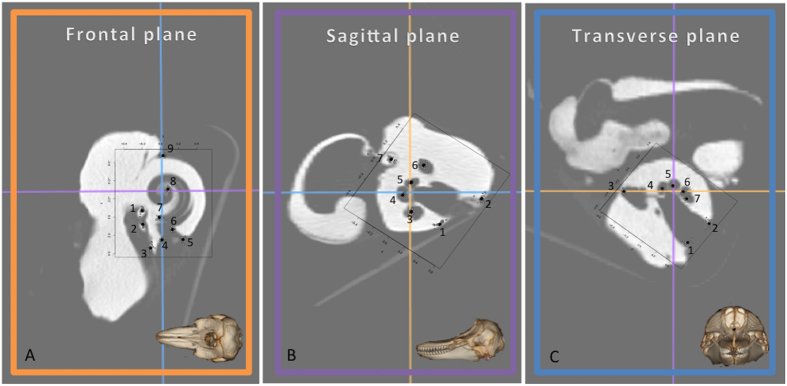
Landmarks (numbered points) for sperm whale cochleae in frontal (**A**), sagittal (**B**) and transverse (**C**) view. Note that the orientation of the T-P complex in sperm whales is slightly rotated in comparison with other odontocetes. See the Table 3 for detailed landmark descriptions.

The morphological assessment of sperm whale ear structures was consistent with descriptions of other members of toothed whales^21^,^31^. No evidence of fractured or recently healed tympanic-periotic bone complexes could be detected in our investigation. Using modern CT scan technology we were able to identify and place 23 landmarks (LMs) on important anatomical features (Fig. 3 and Table 3). No apparent outliers were observed in the studied specimens. General measurements revealed a relatively large range in cochlear capsule length (34–40 mm), width (29–32 mm) and height (22–24 mm). These observations were also confirmed by the study of centroid size (CS: 25–34 mm) extracted from the geometric morphometric analysis (Table 4). The 24 sperm whale cochleae (left and right) showed inter-individual variations for shape (ANOVA, *p* = 0.001) and size (ANOVA, *p* = 0.001) for all studied planes, but no directional asymmetry was detected between the left and the right ears for shape (ANOVA, *p* > 0.05) and size (ANOVA, *p* > 0.05).

### Geometric morphometric analyses

**Figure 4:**
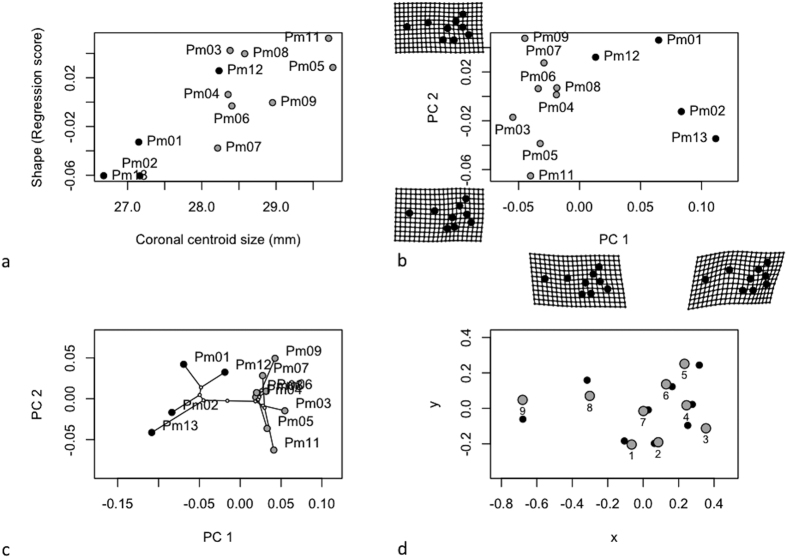
Analysis of cochlear shape in frontal view (**a**) Allometry of cochlear shape represented by the regression of shape values (common allometric component) at function of the centroid size (**b**) Morphospaces defined by PC axes illustrating morphological diversity in sperm whale cochleae. Each point represents the average cochlea shape of an individual. Axes are principal component 1 (PC1) and principal component 2 (PC2) of the average scores from principal components analyses of mean Procrustes shape coordinates for each individual, (**c**) the phylomorphospace, a projection of the phenetic tree (UPGMA dendrogram) into the frontal view PC morphospace (**d**) estimated changes in frontal view shape are shown as deformations from the mean shape among the two stranding clusters. The shape differences have been amplified by a factor of two to aid in the description of shape differences and facilitate biological interpretation.

Size and shape of cochleae in frontal view (separates ventral from dorsal) is constrained by the length and height of their cochlear capsule. Comparisons on frontal CS indicated differences in size among stranding clusters (Table 5 top panel). The cochleae from sperm whales of the first cluster (Fig. 4a in grey) were bigger than those of the second cluster (Fig. 4a in black), despite the fact that this second cluster regroups the largest individuals. Allometry explained a small part of cochlea shape variation (R^2^ = 0.238) for the frontal view (Table 5 top panel; Fig. 4a). Variables related to growth, development and life history of the whales (like age, length and weight) could not be associated to shape and size variation of sperm whale cochleae in frontal view (p > 0.05). The first principal component of shape variation (PC1) explained 43.9% of the total variation, while PC2 explained 17.5% (Fig. 4b). Cochlea shapes differed significantly between stranding clusters, occupying different positions in the frontal cochlea morphospace (MANOVA: F_1,11_ = 14.8, p = 0.002). A phenetic covariance matrix was calculated by UPGMA and we tested for the existence of phenetic signals in centroid size and shape. We found significant phenetic signal for both centroid size (K = 1.52, p = 0.001) and shape (K = 1.12, p = 0.001), indicating that our stranding clustering partially determines the intra-specific variation observed in the morphology of the cochleae in frontal view (Fig. 4c). This relationship was also confirmed by the significant correlation between the distance matrix computed from the 31 external morphometric characters and the Procrustes distances for frontal view (Mantel test: r = 0.49, p = 0.01, Fig. 2c). Vector displacements illustrate the shape changes between the cochleae of sperm whales from the two clusters (Fig. 4d). Individuals from cluster 1 (in grey) showed a displacement of the centre of the cochlea (LM 8), a displacement of the junction between the periotic and the tympanic bone (LM 9) and an enlargement of the cochlear aqueduct (LMs 3 & 5) compared to the sperm whales of cluster 2 (Fig. 4d, in black).

**Figure 5:**
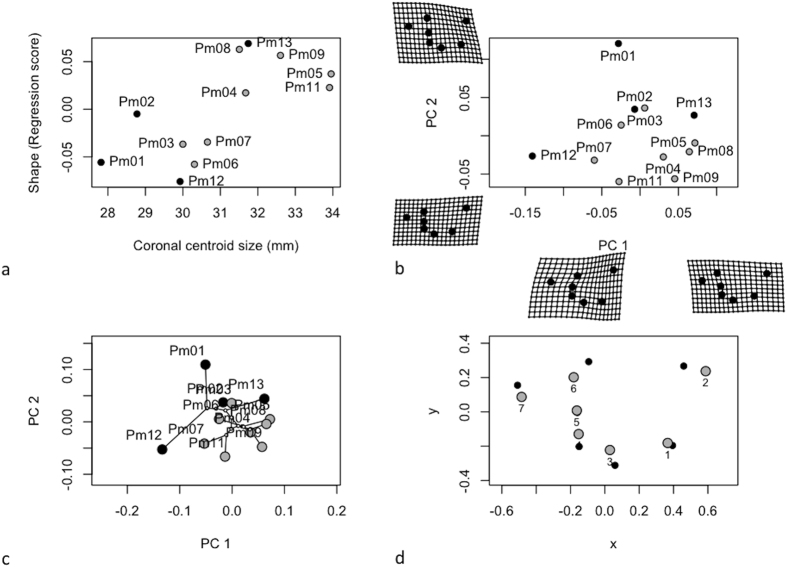
Analysis of cochlear shape in sagittal view (**a**) Allometry of cochlear shape represented by the regression of shape values (common allometric component) as function of the centroid size (**b**) Morphospaces defined by PC axes illustrating morphological diversity in sperm whale cochleae. Each point represents the average cochlea shape of an individual. Axes are principal component 1 (PC1) and principal component 2 (PC2) of the average scores from principal components analyses of mean Procrustes shape coordinates for each individual, (**c**) the phylomorphospace, a projection of the phenetic tree (UPGMA dendrogram) into the sagittal view PC morphospace (**d**) estimated changes in sagittal view shape are shown as deformations from the mean shape among the two stranding clusters. The shape differences have been amplified by a factor of two to aid in the description of shape differences and facilitate biological interpretation.

Length and width of their cochlear capsule constrained the size and shape of cochleae in sagittal view (separates left from right). Individuals from stranding clusters differed on cochlear width and sagittal centroid size (Table 5 middle panel). The cochleae from the largest individuals (cluster 2, Fig. 5a in black) were smaller than those of the first cluster (Fig. 5a in grey). Allometry explained a part of cochlea shape variation (R^2^ = 0.268) for the sagittal view (Table 5 middle panel; Fig. 5a). Variables like age, length and weight (closely related to growth, development and life history of the whales) could not be associated to shape and size variation of sperm whale cochleae in sagittal view (p > 0.05). The first principal component of shape variation (PC1) explained 45.6%, and PC2 29.5% of the total variation (Fig. 5b). Cochleae of sperm whales differed significantly among stranding locations (MANOVA: F_1,11_ = 5.41, p = 0.035). No phenetic signal for both centroid size (K = 1.03, p = 0.097) and shape (K = 0.95, p = 0.061) were observed in sagittal view (Fig. 5c). This non-significant result was confirmed by the Mantel test assessing the correlation between similarities based on external morphology and cochlea shape in sagittal view (r = 0.28, p = 0.07, Fig. 2d). Individuals from cluster 1 (in grey) showed a displacement of the centre of the cochlea (LM 3 & 4), a displacement of the junction between the periotic and the tympanic bone (LM 7) and an enlargement of the cochlear nerve canals (LM 2 & 6) compared to the sperm whales of cluster 2 (Fig. 5d, in black).

**Figure 6:**
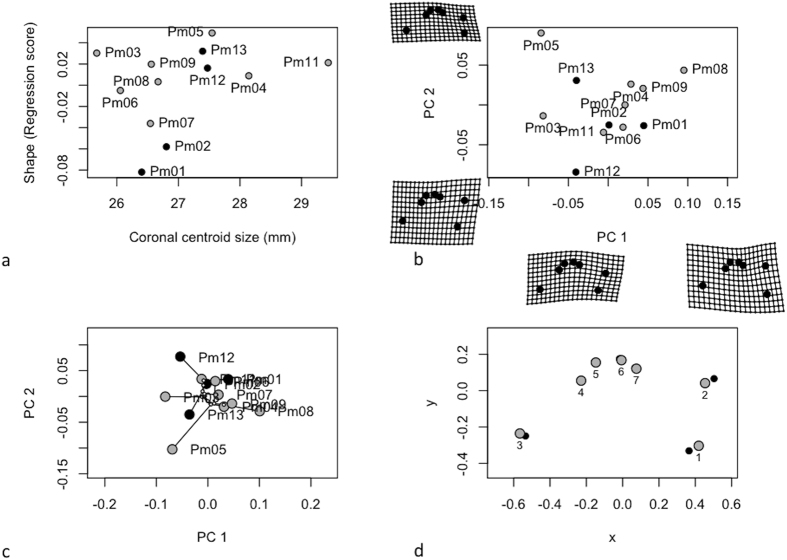
Analysis of cochlear shape in transverse view (**a**) Allometry of cochlear shape represented by the regression of shape values (common allometric component) as function of the centroid size (**b**) Morphospaces defined by PC axes illustrating morphological diversity in sperm whale cochleae. Each point represents the average cochlea shape of an individual. Axes are principal component 1 (PC1) and principal component 2 (PC2) of the average scores from principal components analyses of mean Procrustes shape coordinates for each individual, (**c**) the phylomorphospace, a projection of the phenetic tree (UPGMA dendrogram) into the transverse view PC morphospace (**d**) estimated changes in transverse view shape are shown as deformations from the mean shape among the two stranding clusters. The shape differences have been amplified by a factor of two to aid in the description of shape differences and facilitate biological interpretation.

Size and shape of cochleae in transverse view (separates head from tail) was mainly driven by width and height. No significant differences could be identified among stranding clusters for transverse centroid size (p > 0.05) and no allometric variation was detected (Table 5 bottom panel; Fig. 6a). The first principal component of shape variation (PC1) explained 46.2% of the total variation, while PC2 explained 33.1% (Fig. 6b). Along its transverse view, no shape differences were detected between clusters based on external morphology (p > 0.05). Only small variation of the cochlear nerve canal was observable (LM 1 & 2, Fig. 6d). We found no phenetic signal for both centroid size (p > 0.05) and shape (p > 0.05), indicating that the stranding clustering does not determine the intra-specific variation observed in the morphology of the cochleae in transverse view (Figs 6c and 2e).

## Discussion

Cetacean T-P bone complexes present extreme compactness, density and mineral content, which are functionally supposed to increase the efficiency of ultrasound conduction and to facilitate the bilateral discrimination of sound direction underwater^16^,^17^,^18^,^19^,^20^,^21^,^22^,^23^,^24^,^26^,^32^. However, due to its low organic content, the cetacean tympanic bulla is friable, which make it more susceptible to fracture^33^,^34^. Such fractures might occur when whales have been exposed to a loud acoustic source that was matched to the resonant frequency of the bulla. Information about the sperm whale positions prior to the strandings are missing as well as information on surrounding naval manoeuvres. No evidence of fractured or recently healed tympanic-periotic bone complexes could be detected in our study which could have caused hearing damage to the animals, however, this does not exclude that exposure to noise might have not led to behavioural changes. Nevertheless, a certain heterogeneity of sperm whale ears between individuals became rapidly visually evident during necropsies. The anatomical descriptions found in literature were often made on single specimens and inter-individual variations were not taken into account. For the first time, our study illustrates considerable inter-individual variability in size and morphology of the cochlea in subadult sperm whales. Various factors may explain such a variation among groups of *Physeter macrocephalus*.

One potential factor of variance might be variation related to symmetry. Indeed, skulls of odontocetes are typified by directional asymmetry, particularly in elements associated with the airways and it is assumed that this asymmetry is related to biosonar production^35^. To investigate the degree to which directional asymmetry might contribute to directional cues in sperm whale sound reception, we compared both left and right bony components of cochleae. We could not reveal any asymmetry in the sound reception structures, as the left and the right ear showed a bilateral symmetry for the cochlear size and shape. The lack of asymmetry was also revealed in ears of *Inia geofferensis* and *Delphinus capensis* and additionally performed vibrational analyses suggest that the resonant frequency modes of left and right ears were identical in functional significance for these species^36^. The asymmetry of the T-P bone complexes is apparently not part of the functional component of the odontocete sound-reception apparatus.

The observed inter-individual differences in size and shape of sperm whale cochleae might also be related to differences in size among individuals. The sperm whales that stranded in the North Sea during January and February 2016 were young males sized between 10 and 12 m and aged around 10 to 15 years. However, variation related to age and size would presume that postnatal growth of cochlear structures occurs. No association could be detected between shape and size variation of sperm whale cochleae and variables like age, length and weight, which are closely related to growth, development, and life history of the whales. Based on the collected data to date, postnatal growth of cochlear structures is unlikely^37^. The maximum sizes of the tympanic and periotic bones are already acquired in new-born common dolphins (*Delphinus delphis*), and reached their full mineralization within the first 6 months^33^. No differences were found in tympanic and periotic bone sizes between juvenile and adult bottlenose dolphins (*Tursiops truncatus*) and La Plata dolphins (*Pontoporia blainvillei*)^33^,^37^. Postnatal growth has been described in the anterior spine of the tympanic bulla but not in the periotic bone in *Mesoplodon* species^31^. Only Bisconti (2001) reported significant postnatal growth in the posterior process of the periotic bone in fin whales (*Balaenoptera physalus*)^38^.

A third possibility is that the observed variations are related to the structure of bachelor schools as the morphology of cochleae varied among stranding locations and clusters of sperm whales. Except during breeding seasons, male and female sperm whales are geographically distant. Males leave their cohort between an age of 4 to 21 years and live either solitary or form loose bachelor groups with other males of similar age and size^3^. These groups which live and travel together over years, vary in size and are composed of six to nine individuals but can reach up to twenty^4^. Based on our hierarchical cluster analysis we could classify these individuals in two separate stranding clusters. A first cluster grouping seven sperm whales found on 01/02 in Kaiser-Wilhelm-Koog and an individual (Pm03) stranded two weeks earlier (13/01) in Büsum Süderpiep. This specific individual Pm03 presented an extremely full stomach of Boreoatlantic Armhook Squid (*Gonatus fabricii*) that occurs in the northern Atlantic Ocean from Canada to the Barents Sea, indicating that the animal was foraging in northern waters before it beached. We hypothesize that this animal was the first of a larger group of eight sperm whales that entered into the North Sea mid-January. The other stranding cluster is composed of four animals that stranded on 12/01 on Helgoland and on 03/02 in Büsum.

The shape variations were more pronounced in the frontal and sagittal view than in the transverse one. Size and shape of cochleae in transverse view were mainly driven by width and height and the disposable space inside of the cavities below the brain case where the ears are located possibly limit the variation and expansion of the height of the ears. This might explain the fact that no variation could be revealed in the transverse view. Geographical differences in the middle ear of Guiana dolphins (*Sotalia guianensis*) were found in a recent study between the coasts of northern and south-eastern Brazil, which are consistent with population genetic structure^39^. The traditional morphometrics of the T-P bone complex revealed to be an efficient tool to identify geographic variations in this species. Similarly to this study on *S. guianensis*, our analysis of landmark-based geometric morphometrics suggests that the sperm whales that stranded on German coasts came from distinct groups. Apparently, the cochleae are individually shaped, varying greatly in dimensions and the intra-specific variation observed in the morphology of the cochleae may partially reflect their affiliation to their bachelor school. These regional differences have to be considered in future examinations of samples; these observations could be confirmed by other techniques of morphology, phenology, behavioural ecology, diet and ultimately genetic structure of bachelor schools.

The functional consequences of inter-individual variation of cochlear structure are difficult to define. Size and shape are important components of functional morphology of the T-P bone complex because these factors determine their vibrational parameters^25^,^40^. Generally, an increase of the cochlear size accommodates with more hair cells that respond to sounds^41^. Wannaprasert *et al*. (2015)^14^ showed that the cochlear volume and length are associated with improved low-frequency hearing while the form and shape of the cochlea is not related to an extension of frequency range towards lower frequencies^41^.

## Conclusion

The landmark-based geometric morphometrics was revealed to be an efficient tool to identify inter-individual variations of the tympanic-periotic bone complex of sperm whales. Some shape variation is associated to allometry but a relative amount of shape variation could be attributable to geographical differences and social groups. Our geometric data suggest that the sperm whales that stranded on German coasts came from distinct bachelor schools. Apparently, the cochleae are individually shaped, varying greatly in dimensions and that the intra-specific variation observed in the morphology of the cochleae may partially reflect their affiliation to their bachelor school. Future research might analyse the functional consequences of morphological variation of cochlear structures. There are increasing concerns about the impact of noise on cetaceans and describing the auditory periphery of odontocetes is a key conservation issue to further assess the effect of acoustic pollution.

## Materials and methods

### Specimens

Several mass strandings of sperm whales occurred in the North Sea during January and February 2016. During this period, thirty dead animals were observed along European coasts among them sixteen beached on German coasts (Fig. 1). Twelve animals were necropsied and sampled around 48 h after their discovery (Table 1). A standardised procedure derived from the protocol for necropsies on cetaceans^42^ was used on each carcass.

### Morphometric analysis of external character

Thirty-one external morphometric characters were measured on the twelve specimens of stranded sperm whales (Table 2). Age determination of sperm whales was realised by counting growth layer groups (GLG’s) in the teeth^43^. Weight has been measured or estimated by the following equation (Weight (T) = 0.006648 Length^3.18^)^44^. To determine morphological similarities among individuals, a matrix of pairwise Euclidean distances was calculated from the means of the 31 morphometric traits, and a hierarchical cluster analysis based on this matrix was performed using the unweighted pair-group method with arithmetic average algorithm (UPGMA) (Fig. 2a). The cophenetic correlation coefficient was computed to indicate the degree to which distances in the resulting dendrogram accurately represent the original inter-individual distances^45^. To determine the variables which mainly explained the clustering, we performed a Principal component analysis on the 31 external morphometric characters (Fig. 2b).

During the necropsies sperm whale ears were approached from the ventral side after removal of the lower jaws. The ears sit in cavities below the brain case, located either side of the occipital condyles and behind a large squamosal shield. The ears consist of two dense joined bones about the size of a tightly closed fist. We removed the soft tissue surrounding the ear bones with a knife to find the tympanic-periotic (T-P) bone complex. In sperm whales there is an osseous connection between the ears and the skull, so that the ears had to be cut or levered out of the skull. After removal, the ears were fixed immediately in 10% buffered formalin.

### Computerised tomography imaging

To conduct a comparative analysis of sperm whale ear morphology, we used computerised tomography (CT). Amongst other advantages, CT is a non-invasive technique and allows the information, obtained in a series of slices, to be further rendered in 3D. CT scans of the T-P bone complex of both ears from twelve sperm whales were performed using a BrightSpeed, GE Medical Systems (General Electric). The tympanic-periotic bone complexes were examined for perimortem fractures: such fractures might occur when whales have been exposed to a loud acoustic source that was matched to the resonant frequency of the bulla^33^,^34^.

The ears were scanned in the same orientation in a helicoidal CT with spiral image acquisition, 120 kV voltage, 200 mA/s exposure, a Pitchfactor of 0.5625, 0.625 mm section thickness with a reconstruction advance of 0.31 mm and resolution of 512 × 512 pixels (being the pixel size 0.29296875 × 0.29296875 mm^2^). The images were stored in digital imaging and communication in medicine (DICOM) format and processed using the computer 2D Orthogonal Multiplanar Reconstruction (MPR) software OsiriX Lite^®^. This mode shows three orthogonal planes, the original data set and the major two perpendiculars to it. All the view ports are equal and behave similarly. The lines on each window show the location of the other two orthogonal planes and permit to align perfectly cochleae in the three anatomical planes: dorsal, sagittal and transverse (Fig. 3), so that we can exclude that variations of angle and orientation during the CT scan might affect the presented results. The images were converted to uncompressed TIFF files to preserve greater details. The contours of the cochlear capsule were outlined and using the measurement function bounding rectangle we could determine the length, width and height of the cochleae capsule on these pictures using ImageJ (National Institutes of Health, USA, Ver.1.50 g).

### Geometric morphometric analyses

We quantified shape and size variation of cochlea using landmark-based geometric morphometric methods^46^,^47^,^48^. An extensive introduction to the applications of geometric morphometrics in biology is provided by Zelditch *et al*. (2012)^49^ and Lawing & Polly (2010)^50^. These methods quantify the shape of anatomical objects from the coordinates of repeatable locations, *i.e.* landmarks (LMs). The data acquisition software TPSDig^51^,^52^ was used to digitise landmarks on the scaled TIFF images. The left and the right ears are on opposite sides and represent mirror images. The right ears have been reflected prior to the analysis to allow LMs correspondence. We applied three different morphometric analyses corresponding to each view (*i.e.* frontal, sagittal and transverse views) and thus created separate data files. The LMs were selected in the cochlear spiral to be representative of the spiral shape and its potential variations in different individuals. Nine, seven and seven LMs for cochleae in frontal, sagittal and transverse view were used, respectively. Figure 3 illustrates the LMs configurations on each cochlea view and a detailed description of every LM can be found in Table 3. The LMs were intentionally positioned at key locations that are easily recognisable (such as canals or visible portions of ear bones in the different planes) and were placed at the arithmetic centre of these structures.

For each cochlea view, landmark configurations were optimally aligned using a generalized Procrustes superimposition^53^ using the function ‘gpagen’ of the R-package geomorph^54^. The grand mean was calculated (i.e. the consensus of all specimens), and shape variables were then generated^46^,^55^. The centroid size (CS) was computed as the square root of the sum of the squares of the distances from all LMs to their centroid^56^.

We checked directional asymmetry of the left and the right sperm whale ear to investigate the degree to which directional asymmetry might contribute to directional cues in odontocete sound reception. To do so, shape variation was decomposed into variation among individuals and variation among sides. These components were statistically evaluated using Procrustes ANOVA using the function ‘ bilat.symmetry’ of the R-package geomorph^54^.

We tested the null hypothesis that cochlea shape is unrelated to cochlea size (CS). A Procrustes ANOVA with permutation procedures was performed to assess statistical hypotheses describing patterns of shape covariation with size for a set of Procrustes-aligned coordinates using the function ‘ procD.allometry’ of the R-package geomorph^54^. The results of this function provided the data for plotting allometric curves (Figs 4a, 5a and 6a).

To explore differentiation in cochlea shape across sperm whale groups, we performed a principal components analysis on shape variables to explore inter-individual variation. Deformation grids were used to illustrate shape variation along the principal component axes (Figs 4b, 5b and 6b).

The relative amount of shape and size variation was evaluated, as well as the shape variation associated with variation in centroid size. We quantified the relative amount of shape variation attributable to stranding location and stranding cluster as a factor in a linear model and estimated the probability of this variation, via distributions generated from resampling permutations. This was performed using a Procrustes ANOVA which is implemented in the ‘ procD.lm’ function of the geomorph R-package^54^,^57^. We used Procrustes ANOVA with permutation procedures to assess if variables like age, length and weight (closely related to growth, development and life history of the whales) may influence shape and size of sperm whale cochleae.

The extent to which our classification of individuals based on overall similarity in external morphology (see above UPGMA based on external traits) is translated into variations of shape and sizes of the bony component was also evaluated. We used the previously computed phenetic covariance matrix and tested for the existence of phenetic signals in centroid size and shape using the function ‘physignal’ of the R-package geomorph^54^. In both cases, we evaluated the significance of the observed phenetic signal with 1000 permutations (Figs 4c, 5c and 6c). To confirm this observation, we produced matrices of pairwise Euclidean distances based on the Procrustes distances among mean individual shapes and we performed a hierarchical cluster analysis based on these matrices using UPGMA (Fig. 2c,d,e). We tested the correlation between the distance matrix computed from the external traits and the matrix of the procrustes distances from cochleae shape using a Mantel test (function of the R ade4 Library).

Finally, the shape differences between the cochleae of sperm whales from the two stranding clusters could be visualised graphically, by obtaining the average landmark coordinates for each group and the overall mean, and plotting the differences as vector displacements (Figs 4d, 5d and 6d).

All the analyses were performed in R 3.1.1^58^ using routines in the library geomorph^54^. Statistical significance was accepted at p < 0.05.

## Additional Information

**How to cite this article:** Schnitzler, J. G. *et al*. Size and shape variations of the bony components of sperm whale cochleae. *Sci. Rep.* **7**, 46734; doi: 10.1038/srep46734 (2017).

**Publisher's note:** Springer Nature remains neutral with regard to jurisdictional claims in published maps and institutional affiliations.

## Figures and Tables

**Table 1:** Basic biology data gathered from stranded sperm whales with date of first report (dd/mm/yy), stranding location, necropsy performed, age (y), *measured weight or estimated weight (Weight (T) = 0.006648 Length^3.18^; Lockyer, 1981^44^) and length (m).

Name	Date	Stranding location	Necropsied	Age (y)	Weight (T)	Length (m)
Pm01	12/01/16	Schleswig-Holstein, Helgoland	Yes	13	19.4	12.3
Pm02	12/01/16	Schleswig-Holstein, Helgoland	Yes	13	18.0	12.0
Pm03	13/01/16	Schleswig-Holstein, Büsum	Yes	12	12.5	10.7
Pm04	1/02/16	Schleswig-Holstein, Kaiser-Wilhelm-Koog	Yes	12	11.8	10.5
Pm05	1/02/16	Schleswig-Holstein, Kaiser-Wilhelm-Koog	Yes	11	15.3	11.4
Pm06	1/02/16	Schleswig-Holstein, Kaiser-Wilhelm-Koog	Yes	10	14.8	11.3
Pm07	1/02/16	Schleswig-Holstein, Kaiser-Wilhelm-Koog	Yes	12	10.7	10.2
Pm08	1/02/16	Schleswig-Holstein, Kaiser-Wilhelm-Koog	Yes	10	13.9	10.9
Pm09	1/02/16	Schleswig-Holstein, Kaiser-Wilhelm-Koog	Yes	15	14.4	11.2
Pm10	1/02/16	Schleswig-Holstein, Kaiser-Wilhelm-Koog	No	nd	nd	nd
Pm11	1/02/16	Schleswig-Holstein, Kaiser-Wilhelm-Koog	Yes	12	12.3	10.8
Pm12	3/02/16	Schleswig-Holstein, Büsum	Yes	11	15.3 (15.0*)	11.4
Pm13	3/02/16	Schleswig-Holstein, Büsum	Yes	15	18.0 (18.0*)	12.0

**Table 2:** Principal component loadings for the 31 external morphometric characters measured on each individual of the 12 specimens of stranded sperm whales (PC = Principal component).

	Code	Body measurement	PC1	PC2
	Age	Age	−0.083	**0.222**
**General size measurements - highly dependent of the total length of the animal**	WT	Calculated weight	−**0.262**	0.032
	TL	Tip of snout - notch of fluke (Total length)	−**0.263**	0.015
	T1	Tip of snout - rear rim of fin	−**0.197**	0.074
	T2	Tip of snout - front tip of flipper	−**0.256**	−0.031
	T3	Tip of snout - corner of mouth	−**0.251**	0.044
	T4	Tip of snout - umbilicus	−**0.25**	−0.026
	T5	Tip of snout - front rim of fin	−**0.219**	0.085
	T6	Tip of snout - middle of genital opening/anus	−**0.259**	0,000
	T7	Tip of snout - front of genital opening	−**0.211**	−0.085
	T8	Tip of snout - rear of genital opening	−**0.237**	−0.027
	T9	Tip of snout - anus	−**0.256**	0.014
	T10	Width of skull	−**0.108**	0.053
	T11	Tip of snout - eye	−**0.156**	0.048
	T12	Tip of snout - ear	−**0.241**	0.046
	T13	Tip of snout - front tip of blowhole	−0.009	**0.223**
**Related to the size of the fins**	F1	Width of fluke	−**0.215**	−0.072
	F2	Notch of fluke - front fim of fluke	−**0.198**	−0.01
	F3	Height of fin	**0.138**	−0.021
	F4	Lenght of fin	−0.018	−**0.383**
	F5	Starting point of flipper - tip of flipper	−**0.199**	0.033
	F6	Largest width of flipper	−**0.19**	−0.054
	F7	Outer starting point of flipper - tip of flipper	−0.129	−**0.371**
**Related to blubber thickness**	B1	Dorsal at the level of the caudal insertion of fin	−0.035	**0.079**
	B2	Lateral at the level of the caudal insertion of fin	0.135	**0.199**
	B3	Ventral at the central level of the flippers	−0.036	**0.112**
	B4	Ventral at the level of the caudal insertion of fin	−0.095	**0.223**
**Others**	O1	Width of eye	0.029	−**0.382**
	O2	Rear of eye - ear	−0.083	−**0.285**
	O3	Length blowhole	−0.121	**0.229**
	O4	Width blowhole	−0.023	−**0.431**
		Proportion of Variance	0.447	0.123
		Cumulative Proportion	0.447	0.570

**Table 3:** Descriptions of the landmarks (points) for sperm whale cochleae in frontal (A), sagittal (B) and transverse (C) view.

	Frontal	Sagittal	Transverse
1	Arithmetical centre of the visible portion of stapes	Left extremity of the cochlear nerve window	Left extremity of the cochlear nerve window
2	Arithmetical centre of the visible portion of incus	Right extremity of the cochlear nerve window	Right extremity of the cochlear nerve window
3	Joint between periotic and tympanic (left)	Arithmetical centre of the basal cochlear turn canal	Cochlear aqueduct
4	Joint between periotic and tympanic (right)	Arithmetical centre of the apical cochlear turn canal	Arithmetical centre of the tympanic duct apical cochlear turn
5	Outer side of the cochlear wall	Arithmetical centre of the basal cochlear turn canal	Arithmetical centre of the vestibular duct apical cochlear turn
6	Innerside of the cochlear wall	Arithmetical centre of the facial nerve canal	Arithmetical centre of the vestibular duct basal cochlear turn
7	Begin of interscalar septum	Head of malleus	Arithmetical centre of the tympanic duct basal cochlear turn
8	End of interscalar septum		
9	Posterior intersection between tympanic and periotic bone		

**Table 4:** Length, width, height and centroid size of sperm whale cochlear capsule, results are presented as mean (median) ± sd, min-max.

Length (mm)	Width (mm)	Height (mm)	Centroid size
36.68 (36.62) ± 1.61	31.12 (31.65) ± 1.09	22.93 (22.81) ± 0.92	28.82 (29.08) ± 1.71
34.34–39.89	29.22–32.26	21.62–24.20	24.80–33.95

**Table 5:** Results of ANOVA considering the effects of stranding cluster and stranding location on centroid size (top), shape (middle) and shape while accounting with variation in centroid size (bottom) for all 3 views.

**Frontal plane**							
***Centroid size***	**DF**	**SS**	**MS**	**Rsq**	**F**	**P**	
Stranding cluster	1	5.904	5.904	0.596	12.713	0.002	**
Stranding location	2	0.284	0.142	0.028	0.306	0.759	
Residuals	8	3.715	0.464				
Total	11	9.903					
***Shape***							
Stranding cluster	1	0.029	0.029	0.374	5.365	0.002	**
Stranding location	2	0.005	0.002	0.067	0.483	0.966	
Residuals	8	0.043	0.005				
Total	11	0.078					
***Shape allometry***							
Centroid size	1	0.019	0.019	0.238	3.024	0.006	**
Stranding cluster	1	0.005	0.005	0.067	0.851	0.463	
Stranding location	2	0.010	0.005	0.121	0.767	0.469	
Centroid size × Stranding cluster	1	0.010	0.010	0.126	1.604	0.027	*
Centroid size × Stranding location	1	0.004	0.004	0.054	0.685	0.247	
Residuals	5	0.032	0.006				
Total	11	0.081					
**Sagittal plane**							
***Centroid size***	**DF**	**SS**	**MS**	**Rsq**	**F**	**P**	
Stranding cluster	1	1.364	1.364	0.351	7.311	0.035	*
Stranding location	2	1.026	5.131	0.264	2.751	0.062	.
Residuals	8	1.492	1.865				
Total	11	3.882					
***Shape***							
Stranding cluster	1	0.016	0.016	0.169	1.949	0.082	.
Stranding location	2	0.013	0.006	0.138	0.794	0.498	
Residuals	8	0.065	0.008				
Total	11	0.094					
***Shape allometry***							
Centroid size	1	0.025	0.025	0.268	4.948	0.005	**
Stranding cluster	1	0.003	0.003	0.0322	0.593	0.761	
Stranding location	2	0.013	0.007	0.139	1.287	0.348	
Centroid size × Stranding cluster	1	0.016	0.016	0.166	3.058	0.016	*
Centroid size × Stranding location	1	0.012	0.012	0.123	2.273	0.011	*
Residuals	5	0.025	0.005				
Total	11	0.094					
**Transverse plane**							
***Centroid size***	**DF**	**SS**	**MS**	**Rsq**	**F**	**P**	
Stranding cluster	1	0.010	0.010	0.001	0.009	0.928	
Stranding location	2	2.944	1.472	0.258	1.391	0.271	
Residuals	8	8.466	1.058				
Total	11	1.142					
***Shape***							
Stranding cluster	1	0.005	0.005	0.080	0.927	0.490	
Stranding location	2	0.016	0.008	0.233	1.357	0.193	
Residuals	8	0.047	0.006				
Total	11	0.068					
***Shape allometry***							
Centroid size	1	0.005	0.005	0.080	0.956	0.488	
Stranding cluster	1	0.005	0.005	0.081	0.965	0.432	
Stranding location	2	0.017	0.009	0.254	1.523	0.101	
Residuals	7	0.040	0.006				

Signif. codes: 0 ‘***’ 0.001 ‘**’ 0.01 ‘*’ 0.05 ‘.’ 0.1, DF: degrees of freedom, SS: sums of squares, MS: mean squares, Rsq: R square, F: F statistic, p: corresponding p-value.
